# Confocal Laser Endomicroscopy for the Morphometric Evaluation of Microvessels in Human Colorectal Cancer Using Targeted Anti-CD31 Antibodies

**DOI:** 10.1371/journal.pone.0052815

**Published:** 2012-12-28

**Authors:** Tatiana Cârţână, Adrian Săftoiu, Lucian Gheorghe Gruionu, Dan Ionuţ Gheonea, Daniel Pirici, Claudia Valentina Georgescu, Adriana Ciocâlteu, Gabriel Gruionu

**Affiliations:** 1 Research Center of Gastroenterology and Hepatology, University of Medicine and Pharmacy of Craiova, Craiova, Romania; 2 Department of Mechanical Engineering, University of Craiova, Craiova, Romania; 3 Department of Histology, University of Medicine and Pharmacy of Craiova, Craiova, Romania; 4 Department of Pathology, Emergency County Hospital, Craiova, Romania; 5 Steele Laboratory of Tumor Biology, Department of Radiation Oncology, Massachusetts General Hospital, Harvard Medical School, Boston, Massachusetts, United States of America; The Chinese University of Hong Kong, Hong Kong

## Abstract

**Introduction:**

Numerous anti-angiogenic agents are currently developed to limit tumor growth and metastasis. While these drugs offer hope for cancer patients, their transient effect on tumor vasculature is difficult to assess in clinical settings. Confocal laser endomicroscopy (CLE) is a novel endoscopic imaging technology that enables histological examination of the gastrointestinal mucosa. The aim of the present study was to evaluate the feasibility of using CLE to image the vascular network in fresh biopsies of human colorectal tissue. For this purpose we have imaged normal and malignant biopsy tissue samples and compared the vascular network parameters obtained with CLE with established histopathology techniques.

**Materials and Methods:**

Fresh non-fixed biopsy samples of both normal and malignant colorectal mucosa were stained with fluorescently labeled anti-CD31 antibodies and imaged by CLE using a dedicated endomicroscopy system. Corresponding biopsy samples underwent immunohistochemical staining for CD31, assessing the microvessel density (MVD) and vascular areas for comparison with CLE data, which were measured offline using specific software.

**Results:**

The vessels were imaged by CLE in both normal and tumor samples. The average diameter of normal vessels was 8.5±0.9 µm whereas in tumor samples it was 13.5±0.7 µm (p = 0.0049). Vascular density was 188.7±24.9 vessels/mm^2^ in the normal tissue vs. 242.4±16.1 vessels/mm^2^ in the colorectal cancer samples (p = 0.1201). In the immunohistochemistry samples, the MVD was 211.2±42.9/mm^2^ and 351.3±39.6/mm^2^ for normal and malignant mucosa, respectively. The vascular area was 2.9±0.5% of total tissue area for the normal mucosa and 8.5±2.1% for primary colorectal cancer tissue.

**Conclusion:**

Selective imaging of blood vessels with CLE is feasible in normal and tumor colorectal tissue by using fluorescently labeled antibodies targeted against an endothelial marker. The method could be translated into the clinical setting for monitoring of anti-angiogenic therapy.

## Introduction

Anti-angiogenic therapy has recently raised increasing interest due to the possible implications related to targeted treatment and prognosis stratification for a variety of solid tumors [1]. Nonetheless, the advent of new anti-angiogenic agents into the oncological clinical practice has generated the need for enhanced imaging methods for evaluation of the microvascular network during treatment.

Angiogenesis has been traditionally evaluated by measuring the microvessel density (MVD) on fixed tissue immunostained for a variety of endothelial markers including factor VIII, CD31, CD34 [2] and previous studies have identified microvascular density (MVD) as a potential prognosis factor for a number of solid tumors. CD31, also known as platelet endothelial cell adhesion molecule-1 (PECAM-1) is a pan-endothelial marker for both small and large vessels [2]. Among its many functions CD31 has also been related to the growth and metastatic spread of tumors, being involved in the processes of angiogenesis and vascular permeability [3].

Still, using immunohistochemistry and MVD to estimate angiogenesis in the context of clinical trials has brought up some ethical and practical concerns related to repeated harvesting of biopsies from patients [4]. Functional imaging of tumor vascularity is a promising alternative but most of the clinically available imaging techniques do not have the microscopic resolution required for clinical applications [5]. Recently, confocal laser endomicroscopy (CLE) was developed for the real-time *in vivo* histological analysis of the gut mucosa. High-resolution optical sections in the horizontal plane of the targeted tissue display cellular and subcellular details during ongoing endoscopy [6]. A variety of clinical applications of the technique have already been described in lesions of both the upper and lower gastrointestinal tract, with particular interest on neoplasia, where CLE generates real-time histological diagnosis and targeted biopsies of relevant areas for a higher diagnostic yield than random biopsies [7], [8], [9], [10], [11], [12]. In colorectal lesions, CLE has shown high accuracy in detecting intraepithelial neoplasia based on the pattern of the vascular network and crypt architecture [7].

Currently approved contrast agents for clinical endomicroscopy examinations include dyes with unspecific staining properties such as fluorescein, acriflavine or cresyl violet [13]. However, recent studies have been performed on animal models and human tissue samples using fluorescently labeled antibodies that enabled specific endomicroscopic imaging of epidermal growth factor receptor (EGFR) and vascular endothelial growth factor (VEGF) [14], [15], [16]. By using fluorescein isothiocyanate-labeled antibodies, CLE was able to differentiate expression levels of EGFR in murine xenograft tumors and allowed distinction of human neoplastic and non-neoplastic colorectal tissue based on their EGFR expression patterns [14]. The same group proved that molecular imaging of VEGF is feasible in different rodent models of gastrointestinal cancers. Differences between the fluorescent strength of the VEGF signal of normal and malignant human tissue specimens were also demonstrated [15]. Molecular imaging of EGFR and survivin, an apoptosis inhibitory protein, was also achieved with the probe-based CLE system in esophageal and gastric mucosa of porcine models [16].

The aim of the present study was to evaluate the feasibility of the CLE system for imaging the vascular network of the human colorectal tissue samples of both normal and malignant mucosa using fluorescently labeled anti-CD31 antibodies. Since currently there are no CD-31 markers approved for human use, we have used the CLE imaging technique on fresh non-fixed human biopsy samples stained with anti-CD31 antibodies to test the hypothesis that the CLE system offers appropriate resolution for imaging the tumor vasculature and collecting vascular parameters similar to the currently accepted histopathology techniques.

## Materials and Methods

### Patients

The present feasibility study was performed on endoscopic biopsies obtained from four patients with advanced colorectal adenocarcinomas, which were diagnosed during routine colonoscopy procedures. The patients were subjected to conventional biopsy forceps sampling of paired areas of the normal colonic mucosa (taken at least 10 cm from the tumor) and of the colorectal mass. The study was approved by the Ethics Committee of the University of Medicine and Pharmacy of Craiova. All patients provided their written informed consent after receiving a standard form for biopsy collection via colonoscopy which stated that the biopsy samples were further analyzed by immunohistochemical techniques and used for research purposes. Biopsies were analyzed according to a common staining protocol (below) for conventional histopathology examination and by CLE examinations after staining with specific antibodies.

### CLE Immunostaining Protocol

One paired sample of fresh human biopsies of about 2–3 mm in diameter was harvested during lower endoscopic procedures from both normal and neoplastic colorectal mucosa of each patient and immersed in physiological saline. The fresh biopsies were incubated in the dark with a monoclonal mouse antibody (IgG1, clone MEM-05, 1∶10 dilution) directed against human CD31/PECAM-1 (Exbio, Prague, Czech Republic) and labeled with Alexa-Fluor 488 for 1 hour at 37°C, and rinsed with physiological saline to remove any unbound reagent. Samples were placed on glass slides and immediately imaged by CLE for vascular structures. For optimizing the technique, different antibody dilutions in 1% bovine serum albumin in PBS (BSA, Sigma-Aldrich, St. Louis, Missouri, USA), ranging from 1∶5 to 1∶30, were previously tested on fresh, non-fixed tissue samples and imaged with an automated fluorescence microscope (Nikon 90i, Tokyo, Japan). A time frame of approximately 60 minutes that allows binding of the antibodies to their target and sample imaging without tissue degradation was also established.

### CLE Imaging

In this study, we used the CLE system which integrates a miniature confocal microscope into the distal tip of a conventional flexible endoscope (EC-3870 CIFK, Pentax, Tokyo, Japan). The confocal lens at the distal tip is slightly advanced outside the endoscope, allowing targeted scanning of the structures. During the scanning, the laser delivers an excitation wavelength of 488 nm with a maximum laser power output of ≤1 mW at the surface of the tissue which is controlled by the user during the examination for optimal imaging contrast. The maximum depth of imaging is 250 µm from the surface of the mucosa. The resulting optical sections have a lateral resolution of 0.7 µm for a 7 µm thick slice (z-axis) and a field of view of 475×475 µm.

The endomicroscope was mounted onto a fixed frame and the biopsies were placed on histology glass slides, in direct and gentle contact with the distal tip of the confocal laser endomicroscope. The images were captured by pressing a foot switch pedal and were digitally stored on the system’s hard drive as grey-scale images (150–250 for each biopsy sample) for later download and processing.

### Vascular Network Measurements

**Figure 1:**
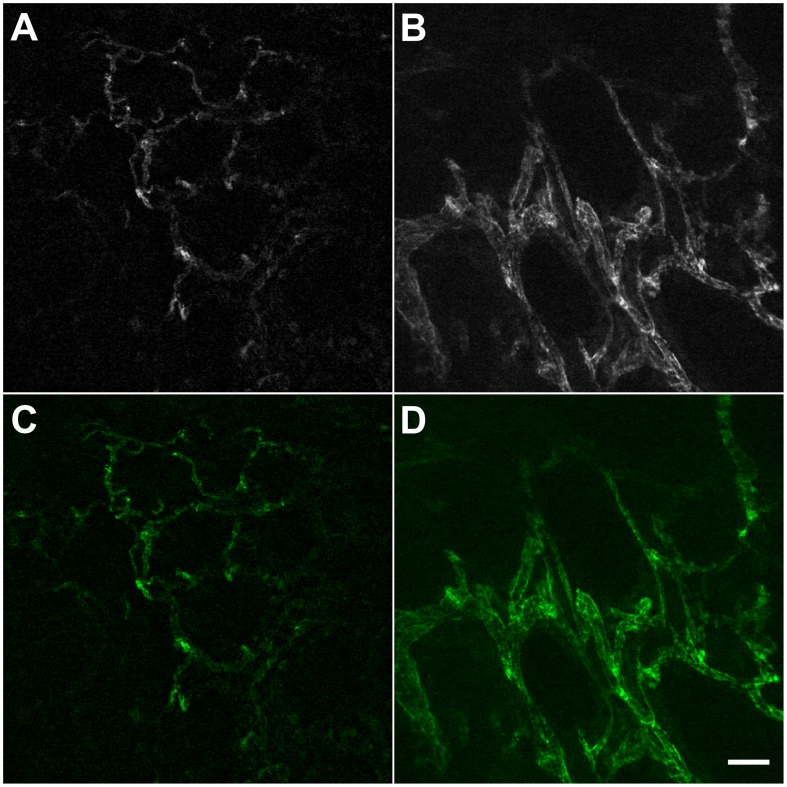
*Ex-vivo* CLE imaging with AF488-anti-CD31-antibodies. In the normal mucosa, the vessels are organized in a hexagonal network (A); the corresponding area in the tumor shows more dilated, irregularly shaped vessels, with varying diameters along their length (B); the same optical sections are shown with added color overlay (C, D). *Scale bar is 50 µm.

We analyzed the CLE images by using Image J (National Institutes of Health, Bethesda, Maryland, USA). Five images with the strongest fluorescent signal and a good display of the vascular network were chosen from each tissue sample for the analysis of the vascular structures. For a more clear distinction of the vessels, a color overlay was added to match the fluorescent contrast agent (Figure 1). The straight line tool was used to manually measure the diameter of each vascular segment between either two branching points or a branching point and a loose end. Each vascular segment was labeled and counted for the vascular density. The results were exported in an Excel file and reported as the mean ± standard error (SE) for each chosen image and individual case. The measurements for diameter and vascular density were performed independently by two different operators who were blinded to each other’s findings and to the pathology report. The results were reported as the mean of the two measurements.

### Conventional Immunohistochemical Staining

Correspondent biopsy samples from colorectal tumors were placed in 4% neutral buffered formalin for fixation and paraffin embedding. Conventional pathology diagnosis was based on H&E staining, while immunohistochemical staining of sections for CD31 was done according to the manufacturer’s indications (Dako, Glostrup, Denmark). Briefly, after citrate buffer-mediated antigen retrieval, sections were cooled to room temperature and incubated for 30 minutes in 1% hydrogen peroxide. The sections were next washed in PBS, followed by a blocking step of 30 minutes in 1% skim milk. The anti-CD31 primary antibody (IgG1, clone JC70A, mouse anti-human, Dako, Glostrup, Denmark) was added diluted as 1∶100, and the slides were incubated overnight at 4°C. Next day, the slides were washed, the signal was amplified utilizing a peroxidase–EnVision polymer-based species-specific secondary detection system (Dako, Glostrup, Denmark), and then detected with 3,3′-diaminobenzidine (DAB, Dako,Glostrup, Denmark). All washing steps were done in 0.1 M PBS, pH 7.2, and the primary antibody was diluted in PBS with 1% BSA. All incubation times were kept constant for all the slides included in the present study. Finally, the slides were coversliped after the Hematoxylin staining.

### Microvessel Density (MVD) Measurement

All microscopic image analysis was performed with a Nikon Eclipse 55i microscope coupled to a 5 Mp color CCD camera (Nikon, Tokyo, Japan) and an image-analysis station based on the Image ProPlus AMS7 software (Media Cybernetics, Bethesda, Maryland, USA). In order to assess the MVDs on the CD-31 stained tumor and correspondent non-tumor tissue sections, the hotspot method was utilized as previously described [17]. In the tumor tissue, the operator used the 10x and 20x objectives to identify the “hot-spots”, i.e. the areas with the highest vascular density. In these areas three to four random images were captured under the 40x objective. In the normal tissue three to four random images were captured under the 40x objective, excluding areas abounding in non-mucosal/inflammatory cells.

The vascular-related CD31 staining areas (region of interest, ROI) were marked directly onto the images by two independent operators and included vessels with an identifiable lumen, as well as single or clusters of non-luminal endothelial cells. Inflammatory cells taking up the stain (i.e. plasmocytes) and red blood cells with no staining around them were excluded. A macro command in Image ProPlus was developed to count the number and measure ROI areas in each image, and the data was reported in an Excel file as MVD and vascular area, respectively. For comparison with other published data, both the CLE vessel density and the MVD determined from immunohistochemistry were normalized to a mm^2^ area.

### Statistical Analysis

Statistical analysis was performed in Microsoft Office Excel® (Microsoft, Redmond, Washington, USA). The results were expressed as averages ± SE of the mean. For the morphometric evaluation, comparison was made between normal and tumor vasculature. To determine the significance of the differences between the vascular measurements, a two-tailed t-test was performed with statistical significance achieved at a p value <0.05.

## Results

### Microvascular CLE Patterns

A specific fluorescent signal was identified with the CLE scope in all biopsies in normal and neoplastic tissue samples. The vessels in normal biopsies formed a hexagonal pattern with homogeneous distribution of diameters characteristic of a normal mucosa as outlined by the selective CD31 staining (Figure 1A and 1C). In tumor samples the vessels appeared dilated and irregular in shape compared to the normal mucosa, with variable diameters along their length (Figure 1B and 1D).

### Morphometric Analysis of Microvessels

**Figure 2:**
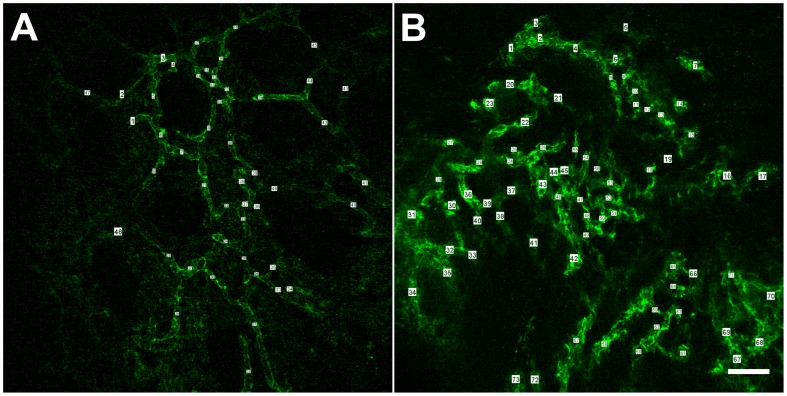
Vascular measurements on CLE images. A vessel was counted and its diameter was measured for each vascular segment between two branching points or a branching point and a loose end. Vascular density was reported per mm^2^. Both vessel diameter and vascular density were increased in the tumor tissue (B) compared to the normal mucosa (A). *Scale bar is 50 µm.

Vessels were counted and their diameters measured between two vascular intersections, for the entire field of view (Figure 2). The average vessel diameter in tumor samples was 13.5±0.7 µm, significantly higher than the vessel diameter in normal colorectal mucosa (8.5±0.9 µm, p = 0.0049). The vascular density was 242.4±16.1 vessels/mm^2^ in tumor tissue samples and 188.7±24.9 vessels/mm^2^ in the normal tissue (p = 0.1201).

### Immunohistochemistry

**Figure 3:**
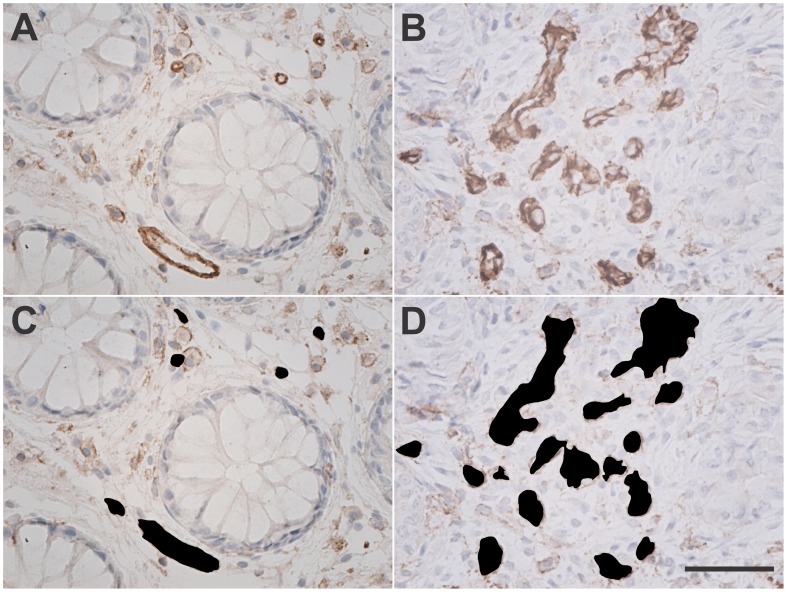
Immunohistochemical staining for CD31. Bright field images of the formalin fixed and paraffin embedded biopsies of normal and primary colonic cancer stained for CD31 showing: blood vessels regularly distributed between the intestinal glands in the normal colonic mucosa (A); multiple irregular vessels in the intratumoral stroma, in malignant samples (B); the same images with vascular areas marked for automated counting (C, D).*Scale bar is 50 µm.

Corresponding biopsies from adjacent tissue to CLE biopsies were prepared for the immunohistochemical study with an anti-CD31 antibody in both normal and colorectal cancer samples. In the normal mucosa, the MVD was 211.2±42.9 while in the malignant tissue it was 351.3±39.6/mm^2^ (p = 0.0637). The vascular area, calculated as the percentage of ROI over the entire image area, was higher in the tumor samples (8.5±2.1%) compared to the normal colorectal tissue (2.9±0.5%), p = 0.0735 (Figure 3). The summary of the vascular parameters measured in the primary colorectal cancer and the corresponding normal mucosa, from immunohistochemistry and CLE samples, is presented in Table 1.

**Table 1:** The vascular parameters obtained with CLE and immunohistochemistry in normal mucosa and colorectal cancer.

	CLE (Mean±SE)		Immunohistochemistry (Mean±SE)	
	Vessel diameter (µm)	Vascular density (vessels/mm^2^)	MVD (vessels/mm^2^)	Vascular area (%)
Normal mucosa	8.5±0.9	188.7±24.9	211.2±42.9	2.9±0.5
Tumor	13.5±0.7	242.44±16.1	351.3±39.6	8.5±2.1
P-value	0.0049	0.1201	0.0637	0.0735

## Discussion

Since the initial hypothesis of the role of angiogenesis in tumor progression formulated by Judah Folkman four decades ago [18], our fundamental understanding of this process has advanced and several anti-angiogenic drugs are either currently in clinical use or under clinical investigation [4]. Nonetheless, there are still many concerns related to selection of the optimal dosage and timing, prediction and monitoring of the patients’ response that limit the use of anti-angiogenic therapy for oncology patients. In the present study, we have used a CLE endoscope to image the vascular pattern from fresh biopsies of normal and malignant colorectal tissue and compared the results with traditional histopathologic techniques.

To our knowledge, this is the first report on histological imaging of vessels obtained with CLE by applying fluorescently labeled antibodies directed against an endothelial marker (CD31). This method generates similar results as the traditional immunohistochemistry, but on fresh non-fixed tissue. Thus, any processing artifact of the biopsies is avoided and imaging of the tissue is performed in its natural state within a short time after biopsy collection which considerably speeds up the diagnosis process. We chose as a molecular target a pan-endothelial marker for labeling of both normal and tumor blood vessels in order to obtain a differential morphometric evaluation. By visual examination, the confocal optical sections showed differences between the vascular patterns of normal and tumoral tissue. The malignant blood vessels looked more dilated and tortuous compared to the regular appearance of the normal vessels. It is a known fact that tumor blood vessels are abnormal in their structure and function. This was also shown by previous CLE classifications of neoplasia following intravenous administration of fluorescein [7], [19]. However this unspecific contrast agent tends to diffuse excessively into the interstitial space due to increased permeability of the pathologic vessels. As a result vascular borders are concealed and their quantitative assessments becomes a challenge. In the present approach, selective staining of vessels with an endothelial marker has overcome this drawback.

With traditional immunohistochemical staining protocols the tissue is typically sectioned in 4 µm thick slices which capture only crossections of blood vessels at different angles. The proposed CLE imaging method allows the display of entire vascular segments in the same image. We were thus able to perform a morphometric analysis on the optical confocal sections that came to validate the qualitative differences already noted between the two vascular beds. We found that tumor blood vessels have a significantly greater average diameter than normal vessels (13.5 µm *vs.* 8.5 µm, p = 0.0049). In tissue samples from the colorectal cancer regions, there was a 28.5% increase in vascular density compared to the normal mucosa (242.4 *vs*. 188.7 vessels/mm^2^). Only one parameter (vessel diameter) reached statistical significance in our study. This could be explained by the small number of samples included in our analysis. Future studies on a larger patient sample are needed to confirm the findings beyond the present proof of concept study.

The vascular parameters obtained with CLE are comparable with the traditional methods and the published literature [20], [21], [22]. The CLE results were validated by a classical immunohistochemical technique which revealed a positive signal for CD31 in both control and malignant samples. There was a similar trend for increased MVD and vascular area in the samples from the primary colorectal cancer compared to the normal mucosa. Previously reported results on human colorectal vascular morphometry vary among different studies due to differences in methodology and processing techniques [20], [21] but overall agree with our results. In a study of the normal colonic microcirculation on corrosion casting a diameter of 10 µm was reported for precapillary arterioles in the mucous plexus [22]. By immunohistochemical staining for CD34, also a pan-endothelial marker, the mean vascular diameter for the normal colonic mucosa was 7.6±1.5 µm [21].

Our proof of concept findings demonstrate the feasibility of the technique and could lay the ground for further research. While this study used a pan-endothelial marker, future similar studies could target other markers for the proliferating endothelium, such as VEGFR2, which are relevant to tumor-induced angiogenesis. The short time frame (one hour between collecting the biopsy and imaging the CD31 marked tissue with CLE) and the use of a standard staining protocol for fresh biopsies samples makes the targeted CLE technique compatible with the clinical setting provided that the equipment is available.

Our present results and prior reports on similar approaches suggest that CLE has the potential for imaging neovascularization *in situ* during live procedures. Since anti-angiogenic drugs tend to normalize tumor vasculature for a short period of time of several days to a few weeks it is desirable to find a way to monitor the vascular network remodeling during this window of opportunity, when the tumor also becomes more responsive to chemotherapy and/or radiotherapy [23]. CLE, alone or in combination with other molecular and cellular biomarkers, could be used for this particular purpose, pending on the discovery of novel targeted contrast agents. As mentioned, recent reports show good preliminary data with fluorescently labeled antibodies targeting EGFR or VEGF, suggesting the feasibility of such immunoendoscopy approaches. Nevertheless, development of human use targeted pan-endothelial markers such as fluorescently labeled anti-CD31 antibodies might represent a valid real-time alternative to the conventional MVD analysis of fixed biopsy samples.

In conclusion, we showed that selective imaging of blood vessels is possible with CLE by using fluorescently labeled antibodies targeting a specific endothelial marker in fresh biopsies. Offline morphometric analysis of the confocal images with additional processing software demonstrated significantly different patterns between normal and malignant vascular networks. In the future this approach could be used for a better selection of patients for clinical trials and a more sensible monitoring of the vascular effects of anti-angiogenic agents in individually tailored therapies.
